# Lifestyle, Nutrition, and Environmental Factors Influencing Health Benefits

**DOI:** 10.3390/ijerph20075323

**Published:** 2023-03-30

**Authors:** Francesca Serio, Antonella De Donno, Giuseppe Valacchi

**Affiliations:** 1 Department of Biological and Environmental Sciences and Technology, University of Salento, 73100 Lecce, Italy; antonella.dedonno@unisalento.it; 2 Department of Food and Nutrition, Kyung Hee University, Seoul 02447, Republic of Korea; gvalacc@ncsu.edu; 3 Animal Science Department, Plants for Human Health Institute, North Carolina State University Research Campus, North Carolina State University, Kannapolis, NC 28081, USA; 4 Department of Biomedical and Specialist Surgical Sciences, University of Ferrara, 44121 Ferrara, Italy

Food is the plants and animals we consume, and nutrition is the way in which food influences bodily wellness. Food is one of the fundamental necessities of existence according to the Academy of Nutrition and Dietetics. Food includes nutrients, which are substances necessary for the regulation of vital processes as well as the development, repair, and maintenance of body tissues.

The World Health Organization (WHO) defines health as “a state of full physical, mental and social well-being and not merely the absence of illness or infirmity” and acknowledges nutrition as a critical component of both health and development. The WHO notes that better nutrition is associated with improved infant, child, and maternal health, stronger immune systems, a decreased risk of non-communicable diseases such as type 2 diabetes and cardiovascular disease, and greater productivity, opening up opportunities to break cycles of poverty and hunger.

Malnutrition, which includes both under- and over-nutrition, poses a serious risk to human health. In particular, undernutrition in young children under the age of five is a worldwide public health issue, with South Asia and Sub-Saharan Africa bearing the heaviest burden.

Egg consumption is extremely low (17%) in Ethiopia due to economic and other factors, with most diets consisting primarily of cereals and lacking in fruits, veggies, and foods derived from animals. The goal of Omer et al.’s research [1] was to determine whether encouraging egg consumption and good poultry husbandry through a child-owned poultry-nutrition intervention would have a beneficial impact on outcomes for growth and development.

In particular, in rural areas, backyard poultry can play a significant role in boosting eggs consumption and thereby help to improve the health and nutritional state of infants and young children. This model of nutrition-sensitive poultry intervention, especially in environments where animal source food intake is low, offers a viable option to treat malnutrition due to its potential for sustainability and results in bettering child growth and development [1].

In addition to nutrition, genetics, surroundings, life cycle, and lifestyle all have an impact on health. Personal dietary preferences are an essential aspect of living, including what and how much a person eats during a meal, how frequently meals are consumed, and how often a person eats out.

According to Guimares’ research, residents had lower odds of regularly consuming vegetables in cities with higher average travel and delay times, and they have lower odds of regularly consuming sugar-sweetened beverages in areas with higher average delay times [2]. This research also revealed that these relationships are stronger in bigger cities, where there are more vehicles on the road and people’s activities typically take place at a greater distance from one another. In turn, the decrease in daily travel time can free up more time to make healthier meals and access foods of higher quality [2].

Finding methods to enhance public health for humans has been the focus of intense research over the past ten years. Studies have primarily concentrated on information about dietary components and nutritional knowledge. Can certain foods help your health? Given that eating is a multidimensional exposure, there are undoubtedly a wide variety of food combos that could be studied.

For instance, recent studies on the primary potential health benefits of wheat-based products show that consuming more whole grains and wheat fiber improves intestinal transit, lowers the risk of obesity and cardiovascular disease, prevents type II diabetes, and lowers the likelihood of developing some types of cancer. Consuming wheat-based products is also linked to a reduced risk of developing chronic diseases. In terms of gluten, protein, amino acids, and the sugar parameters of two wheat cultivars of *Triticum aestivum* L. and barley cultivars of *Hordeum vulgare* L. Moroșan et al. [3] had demonstrated the quantity and quality variations.

For both varieties, significant variations in gluten content, protein, amino acids, and carbohydrates were found. These studies on novel wheat and barley cultivars represent the best use of cross-breeding and selection-based interventions; altering the nutritional profile by enhancing and dosing constituent nutrients can eventually result in the emergence of patient-specific diets tailored to their requirements or nutritional deficiencies. The production of improved cereal cultivars with higher levels of the key nutrients influencing the quality of food products derived from them was emphasized in this research [3].

Human health is influenced by nutrition as well as genetics, climate, and lifestyle, all of which are referred to as “exposome” factors. Lifestyle refers to a person’s daily actions and functions in their job, hobbies, entertainment, and diet. Maintaining a healthful lifestyle enhances general health and happiness.

Physical inactivity is a global pandemic linked to serious chronic diseases, but it can also increase the risk of premature morbidity and mortality, especially when it comes to the onset of conditions such as coronary heart disease (CHD), type 2 diabetes, and breast and colon cancers. This link between physical inactivity and the built environment of the neighborhood, which may affect health outcomes, highlights the significance of neighborhood perception traits such as walkability, sociability, and safety.

According to Parra et al.’s analyses [4], perceived safety, perceived lack of sidewalks or badly maintained sidewalks, and perceived lack of parks and playgrounds were related with patterns of physical activity. 

These results should be taken into account in public health campaigns to encourage physical exercise as a crucial primary preventive action [4].

Early school years should be the beginning of physical exercise for primary prevention, and it should last throughout a person’s lifespan. Early childhood physical education programs that include aerobic activities must be explicitly designated by schools. Recreational activities, such as running, dancing, and swimming, and particular types of resistance training using free weights and/or specialized equipment, should all be included in programs. Children should be encouraged to lead busy lifestyles at home as well.

Promoting health and preventing individuals from developing diseases are the main goals of primary prevention. To stop disease progression to subclinical conditions, especially in susceptible individuals, it is strongly suggested to implemented activities that limit the risk of exposure and/or boost the immune defense of people at risk. The COVID-19 pandemic period, in particular, has had a significant impact on the physical health and psychological well-being of communities. Environmental protection is one of the primary prevention interventions, along with physical activity, that has had a significant impact on both. However, it has also contributed to reinforce the awareness of how important it is to protect us from the spread of infectious diseases and effectively tackle their occurrence.

COVID-19 has undoubtedly emphasized the importance of good hygiene in the fight against disease and has pushed people to practice fundamental hygiene practices, such as as regular hand washing and surface disinfection. Because of this, the worldwide consumption of sanitizers and disinfectants has been rising throughout the COVID-19 pandemic in all high-risk public spaces, including schools, healthcare facilities, and workplaces.

Hypochlorous acid (HOCl) was suggested by Benedusi et al. [5] as a potential candidate for fighting a variety of microorganisms, including spores and viruses. The findings of this research showed that this HOCl treatment can be regarded as a natural, environmentally friendly disinfectant that is effective in eliminating bacteria and viruses and seems to be safe for human health. 

In contrast to genotoxic chemicals, HOCl does not promote the emergence of antimicrobial tolerance.

The majority of the papers in this Special Issue on “Lifestyle, Nutrition, and Environmental Factors Influencing Health Benefits” are concerned with mitigating human health risks, including outdoor exercise, while also considering the positive effects of lifestyle, nutrition, and environmental factors on health conditions. We are still delighted to have met numerous new colleagues who share our passion for the topic and our interest in the impact of food safety on the “One Health” approach, as well as the use of dietary guidelines and nutritional advice as disease prevention strategies, even though they may employ different definitions and approaches to the subject.
